# Aggressive encounters lead to negative affective state in fish

**DOI:** 10.1371/journal.pone.0231330

**Published:** 2020-04-14

**Authors:** Leia Rogers, Ellie Sales, Shokoofeh Shamsi, R. Keller Kopf, Rafael Freire

**Affiliations:** 1 School of Animal and Veterinary Sciences, Charles Sturt University, Wagga Wagga, NSW, Australia; 2 Graham Centre for Agricultural Innovation, Charles Sturt University, Wagga Wagga, NSW, Australia; 3 Institute for Land, Water and Society, Charles Sturt University, Albury, NSW, Australia; Tufts, UNITED STATES

## Abstract

Animals show various behavioural, neural and physiological changes in response to losing aggressive encounters. Here, we investigated affective state, which are emotion-like processes influenced by positive or negative experiences, in a territorial fish following aggressive encounters and explore links to bold/shy behavioural traits. Eighteen 15-month old Murray cod (*Maccullochella peelii*) received three tests in order to determine bold/shy behavioural traits then underwent a typical go/no-go judgement bias (JB) test. The JB apparatus had five adjacent chambers with access provided by a sliding door and fish underwent a training procedure to enter a chamber at one end of the apparatus to receive a food reward but were chased using a net if they entered the chamber at the opposite end. Only one third (N = 6) of fish successfully completed the training procedure (trained fish), and the remaining 12 fish failed to reach the learning criterion (untrained fish). Trained fish housed with a larger aggressive Murray cod for 24 h were significantly less likely to enter intermediate chambers during probe tests compared to control fish, demonstrating a pessimistic response. Trained fish showed “bolder” responses in emergence and conspecific inspection tests than untrained fish, suggesting that shyer individuals were less able to apply a learned behaviour in a novel environment. Our limited sample was biased towards bold individuals but supports the hypothesis that losing an aggressive encounter leads to pessimistic decision-making.

## Introduction

The study of animal emotion is an emerging yet extremely challenging area of research. While objective definitions of emotions are problematic, recent advances have indicated that emotion-like processes, hereafter termed affective states, control some behavioural and physiological responses, regardless of whether these emotions may be subjectively experienced or not [1, 2]. In fish, as in many other vertebrates, losing an aggressive encounter leads to various behavioural changes such as immobility, fleeing and hiding and has important implications for social hierarchies, reproduction and survival [3]. However, it remains unclear whether losing an aggressive encounter influences affective state and associated decision-making. Deciding on the appropriate behaviour after losing an aggressive encounter has important implications for an animal, and may incur a cost such as loss of social rank or access to resources. Behavioural changes in response to losing an aggressive encounter are likely to be influenced by a wide range of environmental and social cues that are likely to be variable and complex, such as the size and fighting ability of the aggressor. Being able to calculate the expected value of a particular response when presented with variable and complex cues, such as following losing of an aggressive encounter, would be an important capacity for any animal [4]. Appraisal of such situations in order to show a particular response has been proposed to influence affective state, which allows for these varied evaluations to be combined to generate an adaptive response to the complex situation [2, 4, 5].

The possibility that animals exhibit heritable and consistent variation in behaviour within individuals and across time and contexts is also referred to as behavioural syndromes or personality [6]. Recently, behavioural traits related to anxiousness and aggressiveness in dogs have been shown to be related to affective state [7] and in humans, behavioural traits related to “boldness”, such as extroversion, are associated with positive affect [8]. One behavioural trait that has been examined in fish probably more than other taxa is the bold/shy axis [9, 10], where boldness is the reaction to a situation perceived as dangerous, such as foraging in the presence of a predator. Behavioural assays to measure bold/shy behavioural traits in fish have been studied extensively, such as the latency to emerge from a hide, latency to eat a novel food, or predator inspection [9, 10].

Recent evidence is emerging indicating that decision-making in fish is influenced by affective state [11–13]. Affective state in animals is often demonstrated using go/no-go judgement bias tests (JB), in which subjects are trained to approach one stimulus to receive a reward and avoid a second stimulus in order to not receive a punisher, or receive a lower value reward. In critical probe tests, subjects are then presented with stimuli intermediate (hence ambiguous) to the two previously learned stimuli. Approaching these ambiguous stimuli is taken to indicate an “optimistic” response and avoiding these stimuli indicates a “pessimistic” response [1, 14]. The previous research on fish behavioural traits and affect suggest that fish may provide an excellent model to examine whether boldness is associated with positive affect, and whether animals varying in bold/shy behavioural traits may respond differently to losing an aggressive encounter. Additionally, tests of JB usually require considerable handling and training [1, 14] and it is not unusual for some individuals to fail to learn the task. To our knowledge information on subjects that do not learn JB tests is rarely provided, yet there is considerable evidence to indicate that cognition and learning are linked to behavioural traits [10, 15], raising the possibility that JB tests may be systematically excluding some individuals.

The aims of this study were to investigate JB in Murray cod (*Maccullochella peelii*) following aggressive conspecific encounters, and to explore the links between cognition and bold/shy behavioural traits. Murray cod were chosen because this species is an apex predator in Southeastern Australian rivers [16–18], and related species use size and aggressiveness to establish and protect a territory [19]. Taking into account territorial behaviour, we predicted that the presence of a larger aggressive cod would induce a negative affective state in a smaller conspecific. This was tested by examining if fish exposed to a larger aggressive cod for 24 h avoided entering intermediate chambers in a JB test compared to isolation-housed control fish. We also predicted that “bolder” fish would show a more positive affective state, and would also be easier to train than “shyer” fish. Lastly, considerable research has indicated how parasites can alter aspects of host fish behaviour post-infection, either in ways that benefit the parasite or more simply by influencing activity levels [20, 21]. For example, *Diplostomum pseudopathaceum* inhabits the eye of trout causing blindness, and subsequently makes fish less mobile and less respondent to its environment [22], which could be misinterpreted with being less bold if the presence of this parasite is overlooked. These findings suggest that some parasites may influence the ability to learn a JB task, either by influencing activity, cognition or “boldness”. We therefore examined all fish for infection with parasites to exclude the possibility of parasites being the cause of any observed variation in behaviour.

## Materials and methods

### Subjects and housing

Eighteen, 15 month-old unsexed Murray cod that were 245±4 mm long and weighed 166±8 g were used in this study. Fish were reared in outdoor earthen ponds by Uarah Fisheries (Grong Grong, New South Wales) and tested in our laboratory after fish had commenced eating, which was no less than 4 days after arrival. We aimed to test fish soon after they started eating to limit the impact of the laboratory conditions on behaviour, and used feeding behaviour as an indicator that fish were settled. Fish were individually-housed in 43 L glass aquariums with a recirculating biological filtration system, and the temperature of the water was maintained in the range of 21°C - 24°C and oxygen levels at 8.6mg/L. Nitrite and nitrate levels, pH and hardness were checked daily using Aquarium test strips (API, Mars Fishcare, New Jersey, USA) and found to be within normal levels for Murray cod. Food (chicken mince) was initially delivered near the mouth of the fish using a 3 ml disposable pipette at least twice a day. Once fish desensitised to the addition of the pipette, it was placed 5–10 cm from the fish and food delivered once the fish approached the pipette. In following feeding sessions, the pipette was placed progressively further away from the fish to encourage a longer approach.

### Bold/shy tests: Emergence, exploration and conspecific inspection

**Fig 1:**
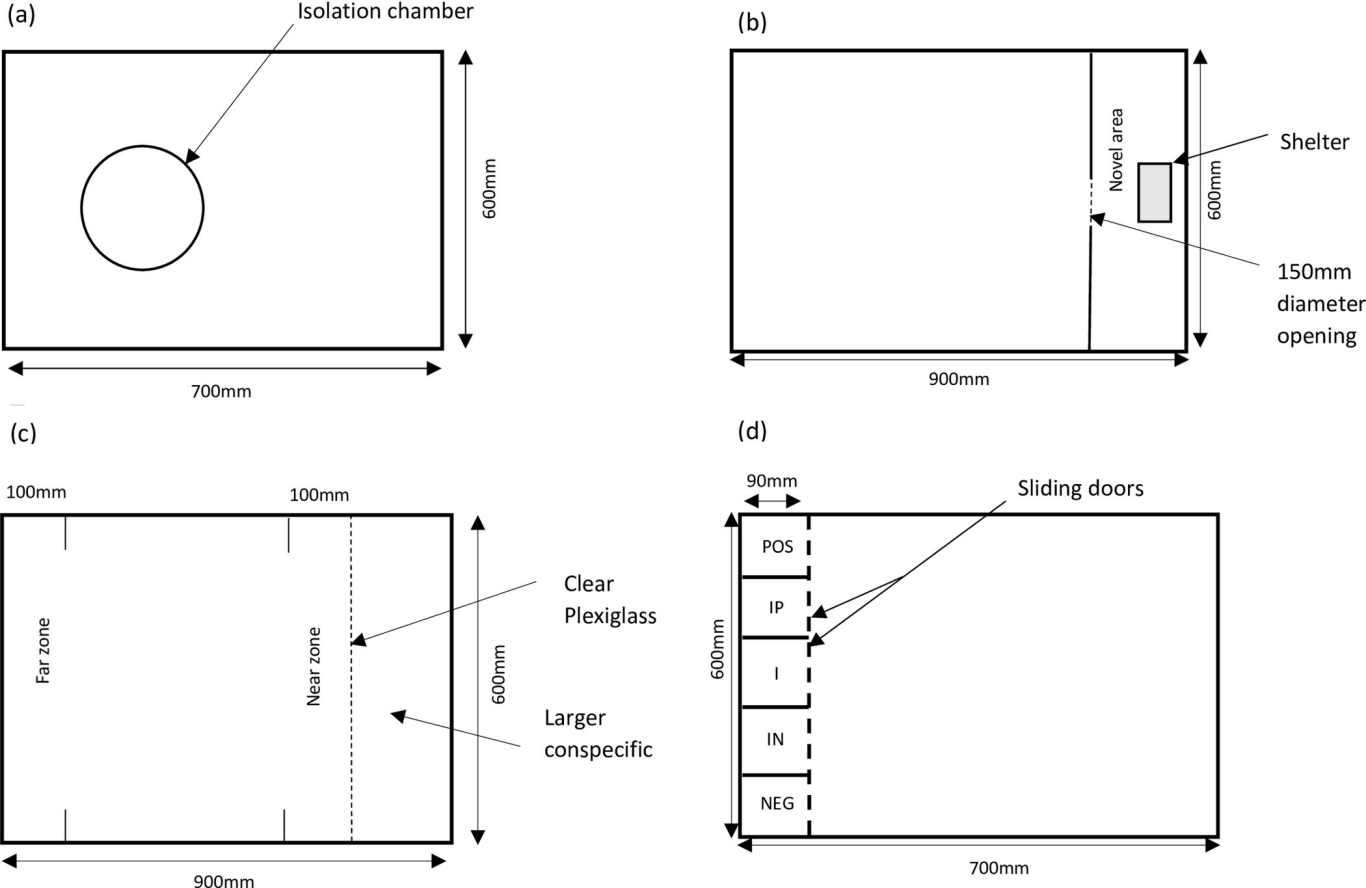
Plan of the aquariums used for (a) emergence, (b) exploration, (c) conspecific inspection and (d) judgement bias testing. Chambers in the JB testing aquarium are referred to as positive, POS; intermediate positive, IP; intermediate, I; intermediate negative, IN and negative, NEG.

After the acclimatisation period, emergence behaviour of fish was examined in a large 200 L tank. Individual fish were placed in an isolation chamber that consisted of a white PVC pipe (250 mm diameter, 600 mm high) placed in the middle of the large tank (Fig 1A). The isolation chamber contained a 250 mm diameter opening that was initially closed with a lid. After the fish was allowed to settle in the isolation chamber for 15 minutes, the lid of the chamber was raised slowly by an experimenter standing out-of-view of the fish so as to minimise disturbance. Latency to emerge was recorded by a video camera (5231 Starlight, Dahua) placed 1.5 m above the isolation chamber, and the test was terminated when the entire fish emerged, or after 15 minutes. After the emergence test, the isolation chamber was removed and the fish allowed to settle for approximately 2 minutes. An opaque screen was then removed by the experimenter revealing a 150 mm opening to a novel area (Fig 1B). We recorded the latency for the entire fish to enter the novel area using an overhead video camera. If the fish did not enter the novel area, the test was ended after 15 minutes. The fish were then returned to the home tank for 24 h before receiving a conspecific inspection test. Fish were moved to a large 200 L tank and allowed to acclimatise for 10 minutes. After this period, an opaque screen was lifted providing visual access to a larger Murray cod (also obtained from the hatchery, and 280 mm long) placed behind a clear Plexiglass screen at one end of the tank (Fig 1C). The total time spent in the near zone, within 10 cm of the larger fish, was recorded for 10 minutes.

### Judgement bias (JB) testing paradigm

Once a fish consistently approached the pipette to feed in the home tanks, and had completed the emergence, exploration and conspecific inspection tests, it was relocated to a 200 L aquarium (585 mm x 700 mm x 600 mm) for JB training and testing. JB training was undertaken in an apparatus consisting of five, 110 mm wide and 90 mm deep opaque Plexiglass chambers that spanned the depth of the water (Fig 1D). Access to each chamber was provided by a sliding door which could be slowly raised by an experimenter standing out-of-view of the fish. Initial training attempted to establish an association between the fish entering a chamber on one side (either left or right) and receiving a positive food reward, and progressed through four stages: 1) eating; 2) approaching the pipette to receive food; 3) opening of chamber sliding door, and approach of pipette in chamber in order to receive food; and 4) as in (3) but the pipette was lowered and food provided after the fish had entered the chamber. Doors were only opened when fish were oriented towards the apparatus, and if they were not, we waited until they re-oriented themselves towards the apparatus before starting a trial. The door opening and the disturbance that this caused in the tank provided the signal to the fish that a trial was starting. Fish were given a food reward if they entered the positive (POS) chamber within 60 s of the researcher opening the door (POS trial). If a fish failed to enter the POS chamber within 60 s (no response), it was given a short break (5–15 minutes) before training was resumed. Immediately after completing 15 correct responses in the POS trials, the sliding door to the chamber on the opposite side (negative, NEG chamber) was opened, and after the fish entered the chamber it was chased with a fish net for 3 s (NEG trial).

**Fig 2:**
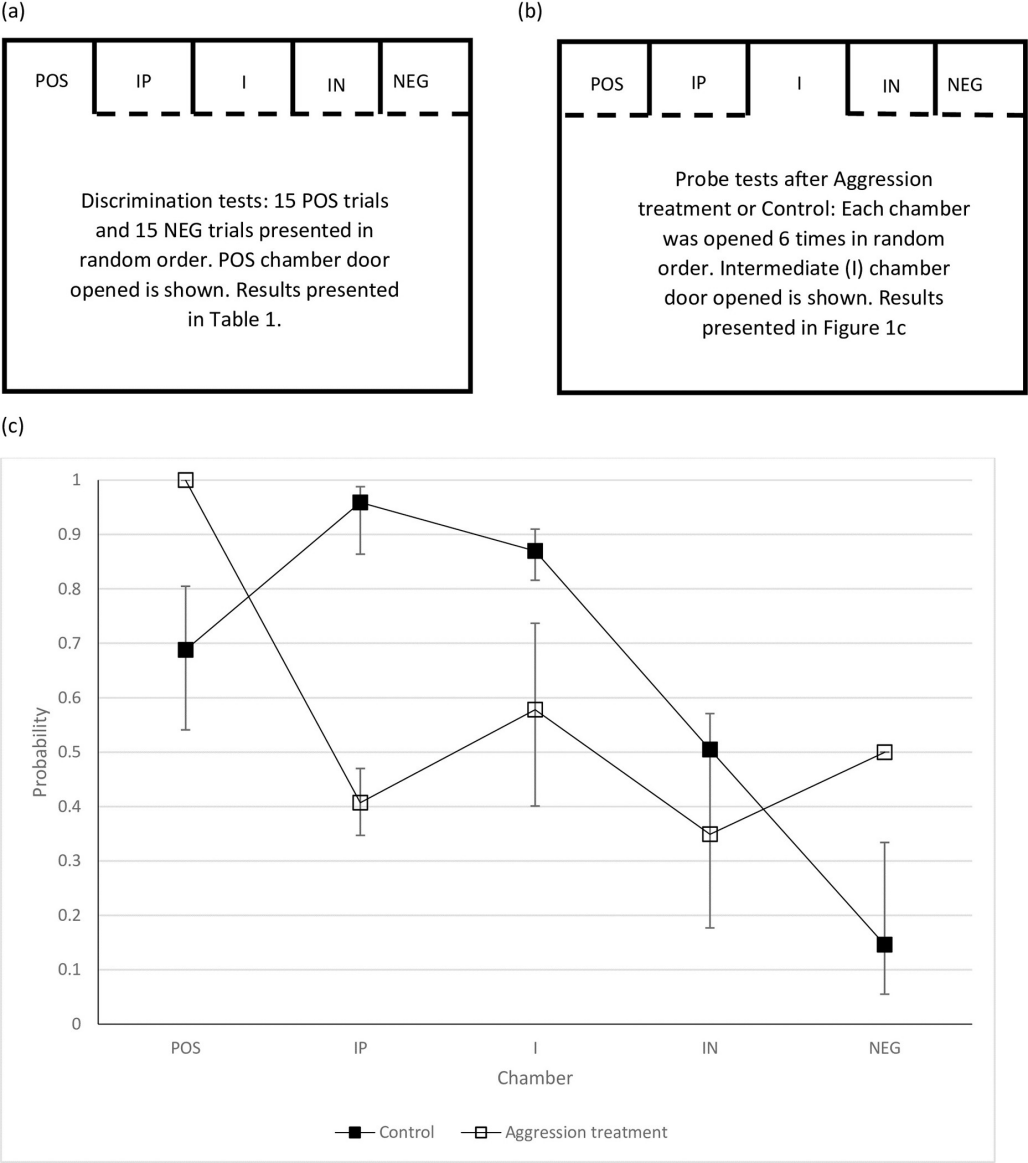
Visual representation of the judgement bias set-up for various tests with Murray Cod: (a) discrimination tests (only positive, POS, and negative, NEG, chambers were opened); (b) probe tests paradigm (in addition to the POS and NEG chambers, IP, intermediate positive; I, intermediate; and IN, intermediate negative chambers were also opened) and (c) Mean predicted probability of fish entering each chamber in probe tests. Aggression treatment fish (white squares, N = 3) were less likely to enter the three intermediate chambers than control fish (black squares, N = 3). 95% Wald confidence intervals are shown.

Fish then undertook 15 POS and 15 NEG trials presented in a random sequence, though starting with a POS trial, and the latency to enter the chamber was recorded (discrimination tests; Fig 2A). Only individuals that showed discrimination between the POS and NEG trials (trained fish, N = 6) progressed to manipulation of affective state and probe tests. Twelve individuals did not enter the POS chamber after three days of training and were returned to the home tanks (untrained fish).

After completing the discrimination tests trained fish remained in the large tank with all chamber doors closed for 24 h. Three trained fish were randomly allocated to two treatment groups after taking into account balancing for side of the tank that the POS trials were presented. Affective state was manipulated by the addition of a larger aggressive conspecific, also obtained from the hatchery and >280 mm long for 24 h (Aggression treatment, N = 3). The control involved housing fish in isolation for 24 h (Control, N = 3). Fish in the Aggressive treatment were observed continuously for the first hour, and at regular intervals thereafter to check for signs of injuries. Following treatment, judgement bias testing consisted of 30 probe trials where one of five doors was opened according to a random sequence, with each door being opened a total of 6 times (though the first trial was a POS trial; Fig 2B). The intermediate chambers (intermediate positive, IP; intermediate, I; intermediate negative, IN; Fig 2B) were not reinforced or punished. Whether or not the fish entered the opened chamber was recorded and the trial ended when the fish either entered a chamber, or after 60 s.

At the end of all behavioural tests fish were euthanised by placing in an ice slurry of 1:1 ice and water and examined for infection with parasites according to standard protocols [23]. Animal use and all procedures were approved by Charles Sturt University’s Animal Care and Ethics Committee (approval number A18099) according to the standards and procedures of the Australian Code for the Care and Use of Animals for Scientific Purposes.

### Statistical analysis

Performance (time; s) in tests of behavioural traits between trained (N = 6) and untrained fish (N = 12) was compared using a t-test, or a Mann-Whitney test if the data did not meet assumptions for parametric analysis. One untrained fish did not receive the emergence and exploration test due to experimenter error. We compared the performance of individual fish in the three tests of behavioural traits using a Spearman rank order correlation.

The binary response to the probe trials (enter chamber = 1, not entering chamber = 0) was analysed using the Generalized Estimating Equations (GEE) function in SPSS (IBM, version 25). The GEE were used to account of the repeated JB trials (N = 30) undertaken on each trained fish (N = 6). The repeated component was fish identity and nested within treatment. The fixed factor was treatment (Aggression, Control). In order to examine the possibility of a pessimistic judgement bias, the first model included the three intermediate chambers (IP; I; IN). In order to examine if the treatment influenced motivation to respond, a second analysis was undertaken comparing the effect of treatment on the outcomes of the POS and NEG trials, again with fish identity nested within treatment as the repeated measures component. For both of the latter two tests, model-predicted probabilities are presented.

## Results

### Judgement bias training and testing

All fish learnt to approach the pipette to feed in the home tanks within 2 days of starting the feeding training, but 12 of these fish did not show this behaviour in the JB testing tanks even after 3 days of training. In contrast, six fish approached the pipette to feed soon after being moved into the JB testing tank, and in discrimination tests, all six of these fish were faster at entering the POS chamber than the NEG chamber (Trained fish; Table 1). During Aggression treatment, fish were observed chasing each other during the first 30 minutes, and both fish attempted to bite each other, with between 4 and 13 bites given or received during this time. Fish were observed and inspected frequently during this time to ensure that there were no significant threats to their physical condition or welfare. Thereafter aggression was unidirectional instigated by the introduced aggressor, and consisted mainly of the aggressor fish swimming towards the other fish which would retreat (termed a threat). Threats persisted throughout the 24 h treatment and the JB trained fish was displaced to nearer the surface for most of the remaining time.

**Table 1:** Results of discrimination tests showing the mean time (±SE) taken to enter the opened chamber in 30 randomly presented positive (POS) food reward and negative (NEG) chasing trials.

Trained fish	POS trial (s)	NEG trial (s)	Treatment	Side of POS chamber
**1**	20.7±5.0	56.0±2.2	Aggression	Left
**2**	14.7±4.8	59.5±0.5	Control	Right
**3**	37.9±4.7	52.5±4.0	Control	Right
**4**	24.3±6.7	57.8±2.1	Control	Left
**5**	23.6±4.4	51.2±3.9	Aggression	Right
**6**	28.5±4.9	54.8±2.5	Aggression	Right

We explored possible changes in affect by comparing the responses of Aggression treatment and Control fish. The number of times fish entered intermediate chambers varied between treatments (χ^2^ = 5.9, df = 1, P = 0.015; Fig 2C). Aggression treatment fish were 12 times less likely to enter intermediate chambers than Control fish (Odds ratio = 12.6, χ^2^ = 15.1, P<0.001). The effects of chamber (χ^2^ = 5.15, df = 2, P = 0.076) or the treatment/chamber interaction (χ^2^ = 5.0, df = 2, P = 0.081) were not significant. All six trained fish were significantly more likely to enter a POS chamber than a NEG chamber during the probe testing phase (χ^2^ = 5.4, df = 1, P = 0.02; Fig 2C), indicating that they continued to discriminate between the POS and NEG chambers. Interestingly, treatment had a significant effect on whether fish entered the POS and NEG chambers (χ^2^ = 5.8, df = 1, P = 0.016; Fig 2C). Aggression treated fish were over 5 times more likely to enter a POS or NEG chamber than Control fish (Odds ratio = 5.9, χ^2^ = 10.3, P = 0.001). Again, the interaction between chamber and treatment was not significant (χ^2^ = 2.3, df = 1, P = 0.13).

### Comparison between trained and untrained fish

**Fig 3:**
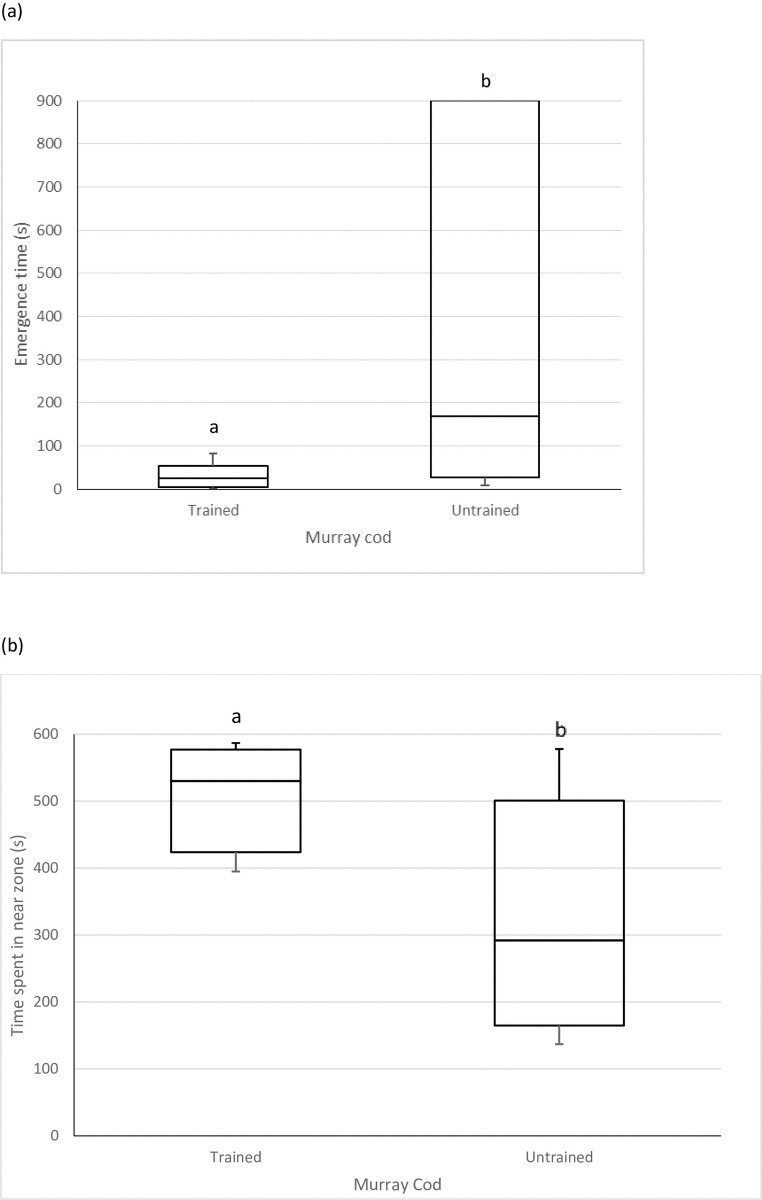
(a) Time taken to emerge from the isolation chamber in the emergence test and (b) time spent in the near zone in the social inspection test by trained and untrained fish. Different letters indicate statistical significance at P<0.05. Range, interquartile range and medians are shown.

Trained fish emerged from the isolation chamber faster (Mann-Whitney, U = 10, N = 17, P = 0.020; Fig 3A) and spent more time in the near zone in the conspecific inspection test than untrained fish (t-test, t = 2.5, df = 10, P = 0.033; Fig 3B). There was no difference in the time taken to explore the novel area between trained and untrained fish (the combined mean latency to enter novel area was 270±70 s; Mann-Whitney, U = 26, N = 17, P = 0.53). However, the time taken to emerge from the isolation chamber in the emergence test, and to explore the novel area in the exploration test, were correlated (Spearman, r = 0.53, N = 17, P = 0.029; though this finding would be NS if a Bonferroni correction for three simultaneous tests was applied). No other significant correlations between the three tests of behavioural traits were found. The most prevalent parasite found was protozoan cysts on the gills, with two fish having over 100 cysts, and *Lernaea* was only found on one fish. There was no indication that gill parasites varied between trained and untrained fish (3/6 trained and 4/12 untrained fish had gill cysts). However, the number of gill parasites was negatively correlated with latency to enter the novel area in the exploration test (Spearman r = -0.49, P = 0.048), but not significantly correlated to response times in other tests.

## Discussion

Our findings show a pessimistic judgement bias in a territorial fish following an aggressive encounter. Comparison of the likelihood of fish entering the POS and NEG chambers in probe tests did not support the possibility that the observed pessimistic response in Aggression treatment fish could have been due to motivational or other factors. Although all our subjects (N = 18) learnt to approach a pipette to feed in the home tanks, 12 fish failed to show this response in the JB testing tanks even after 3 days of trials, and could not be trained. The finding that trained fish emerged from an isolation chamber faster and spent more time near a larger conspecific than untrained fish suggests that fish trained in the JB task were bolder than untrained individuals. Unfortunately, the inability to train shy fish in the JB test limited our ability to further explore the links between bold/shy behavioural traits and affective state.

The change in affect following losing an aggressive encounter contributes to the emerging evidence of affective states being involved in decision-making in fish [11–13]. In fish, losing an aggressive encounter leads to a wide range of behavioural changes, which can be grouped into proactive (e.g. escape) and reactive (e.g. hiding) behaviours [3]. The findings presented here suggest that these responses may be controlled by changes in affective state, and future work could examine exactly how affect is linked to proactive and reactive responses. Additionally, losing aggressive encounters leads to a rise in corticosteroids in fish [24–26] which has been shown to be related to pessimistic judgement biases in rats [27, 28] and chickens [29]. Whether corticosteroids mediate the observed pessimistic judgement bias in Murray cod remains to be confirmed, but the present results are in agreement with the involvement of corticosteroids in pessimistic judgement biases.

Our observations of aggressive displacement, even in juvenile Murray cod, contributes empirical evidence highlighting the territorial nature of this species. The chasing, biting and displacement by an intruder was consistent with field observations of territorial behaviour of eastern freshwater cod (*Maccullochella ikei*) in rivers [19], suggesting that changes in affective state may be an important part of maintaining social hierarchies in wild fish. Additionally, the protocol for examining affective states in fish presented here could be used to examine other pressing questions, such as whether injury is accompanied by a negative affective state in fish [30]. Likewise, hatchery-reared fish including Murray cod often show considerable aggression when in captivity [31], raising concern for their welfare. As the go/no-go JB paradigm is widely used in welfare assessment [1, 14], future research on the welfare challenges fish encounter in commercial systems, laboratories or other environments could utilise this approach.

Our attempt to examine the links between bold/shy behavioural traits and affective state was limited by the failure of “shyer” fish to learn the JB test, reducing both the number of fish in our JB test sample and the variation in behavioural traits of these fish. Our findings at first sight appear to support previous work showing that bold rainbow trout (*Oncorhynchus mykiss*) learned a simple conditioning task faster than shy fish [15]. The untrained fish in our study learnt to approach the pipette to feed in the home tanks just as quickly as trained fish, within 2 days, but did not show this behaviour in the JB testing tanks. This suggests a failure of shyer fish to transfer learning in one environment to a new environment, rather than a difference between trained and untrained fish in learning ability *per se*. Future tests of JB with fish should consider the subject’s experience and familiarity with the testing tanks so as to provide all subjects with an equal opportunity to learn the JB test.

A critical aspect of the judgement bias test is that a pessimistic response should not be accompanied with an avoidance of POS and NEG chambers. Comparison of the likelihood of fish entering the POS and NEG chambers in probe tests did not support the possibility that the observed pessimistic response in Aggression treatment fish could have been due to motivational or other factors. Instead, and rather unexpectedly, Aggression treatment fish were more likely to enter the NEG and POS chambers than control treatment fish. To our knowledge the Aggression treatment-induced increase in response to the unambiguous locations in JB tests has not been reported previously, and may be related to the emotion manipulation and JB testing occurring in the same tank since, normally with terrestrial animals, the emotion manipulation and JB testing is undertaken in different enclosures. Lastly, although in this instance the number of parasites did not vary significantly between trained and untrained fish, parasites in the gill, particularly when occurring in large quantity, usually impair the host’s ability to extract oxygen which is known to reduce swimming activity in fish [32] and may influence latency to respond in behavioural tests. Whether the negative correlation between the latency to enter a novel area and gill parasite numbers presented here was related to the impaired function of the gills remains to be confirmed, but this latter finding supports our assertion that an assessment of fish parasites and health is important to avoid incorrectly assigning variation in behaviour as indicative of stable behavioural traits.

In conclusion, Murray cod showed a negative affective state following an aggressive encounter. Two-thirds of our subjects failed to learn the JB test, and tests of behavioural traits indicated that these fish were shy compared to individuals that were able to learn the JB test. Further studies exploring affective states in fish should utilise behavioural assays that can be completed by bold and shy individuals to eliminate sample bias.

## Supporting information

S1 File Data file. (CSV)

## References

[1] Harding EJ, Paul ES, Mendl M. Animal behaviour: cognitive bias and affective state. Nature. 2004;427(6972): 312 10.1038/427312a 14737158

[2] Paul ES, Harding EJ, Mendl M. Measuring emotional processes in animals: the utility of a cognitive approach. Neurosci Biobehav Rev. 2005;29(3): 469–491. 10.1016/j.neubiorev.2005.01.002 15820551

[3] Butler JM, Whitlow SM, Roberts DA, Maruska KP. Neural and behavioural correlates of repeated social defeat. Sci Rep. 2018; 8(1): 6818 10.1038/s41598-018-25160-x 29717159 PMC5931592

[4] Mendl M, Burman OH, Paul ES. An integrative and functional framework for the study of animal emotion and mood. Proc R Soc B. 2010;277(1696): 2895–2904. 10.1098/rspb.2010.0303 20685706 PMC2982018

[5] Trimmer P, Paul E, Mendl M, McNamara J, Houston A. On the evolution and optimality of mood states. Behav Sci. 2013;3(3): 501–521. 10.3390/bs3030501 25379252 PMC4217599

[6] Dingemanse NJ, Dochtermann NA, Nakagawa S. Defining behavioural syndromes and the role of ‘syndrome deviation’ in understanding their evolution. Behav Ecol Sociobiol 2012;66(11): 1543–1548.

[7] Barnard S, Wells DL, Milligan AD, Arnott G, Hepper PG. Personality traits affecting judgement bias task performance in dogs (*Canis familiaris*). Sci Rep. 2018;8(1): 6660 10.1038/s41598-018-25224-y 29703989 PMC5924375

[8] Marshall GN, Wortman CB, Kusulas JW, Hervig LK, Vickers RR Jr. Distinguishing optimism from pessimism: Relations to fundamental dimensions of mood and personality. J Pers Soc Psychol. 1992;62(6): 1067.

[9] Toms CN, Echevarria DJ, Jouandot DJ. A methodological review of personality-related studies in fish: focus on the shy-bold axis of behavior. Int J Comp Psychol. 2010;23(1): 1–25.

[10] Sneddon LU. The bold and the shy: individual differences in rainbow trout. J Fish Biol. 2003;62(4): 971–975.

[11] Millot S, Cerqueira M, Castanheira MF, Øverli Ø, Martin CI, Oliveira RF. Use of conditioned place preference/avoidance tests to assess affective states in fish. Appl Anim Behav Sci. 2014;154: 104–111.

[12] Cerqueira M, Millot S, Castanheira MF, Félix AS, Silva T, Oliveira GA et al Cognitive appraisal of environmental stimuli induces emotion-like states in fish. Sci Rep. 2017;7(1): 1–10. 10.1038/s41598-016-0028-x 29030568 PMC5640617

[13] Laubu C, Louâpre P, Dechaume-Moncharmont FX. Pair-bonding influences affective state in a monogamous fish species. Proc Roy Soc. 2019;286(1904): 20190760. 10.1098/rspb.2019.0760 PMC6571461 31185864

[14] Bethell EJ. A “how-to” guide for designing judgment bias studies to assess captive animal welfare. J Appl Anim Welf Sci. 2015;18(sup1): S18–S42. 26440495 10.1080/10888705.2015.1075833

[15] Sih A, Del Giudice M. Linking behavioural syndromes and cognition: a behavioural ecology perspective. Philos T Roy Soc B. 2012:367(1603): 2762–2772. 10.1098/rstb.2012.0216 PMC3427552 22927575

[16] Ebner B. Murray cod an apex predator in the Murray River, Australia. Ecol Freshw Fish. 2006;15(4): 510–520.

[17] Jones MJ, Stuart IG. Movements and habitat use of common carp (*Cyprinus carpio*) and Murray cod (*Maccullochella peelii peelii*) juveniles in a large lowland Australian river. Ecol Freshw Fish. 2007;16(2): 210–220.

[18] Koehn JD, McKenzie JA, O’Mahony DJ, Nicol SJ, O’Connor JP, O’Connor WG. Movements of Murray cod (*Maccullochella peelii peelii*) in a large Australian lowland river. Ecol Freshw Fish. 2009;18(4): 594–602.

[19] Butler GL, Rowland SJ. Using underwater cameras to describe the reproductive behaviour of the endangered eastern freshwater cod *Maccullochella ikei*. Ecol Freshw Fish. 2009;18(3): 337–349.

[20] Thomas F, Poulin R, Brodeur J. Host manipulation by parasites: a multidimensional phenomenon. Oikos. 2010;119(8): 1217–1223.

[21] Poulin R. Parasite manipulation of host personality and behavioural syndromes. J Exp Biol. 2013;216(1): 18–26. 23225863 10.1242/jeb.073353

[22] Klemme I, Karvonen A. Learned parasite avoidance is driven by host personality and resistance to infection in a fish–trematode interaction. Proc Roy S B. 2016;283(1838): 20161148. 10.1098/rspb.2016.1148 PMC5031654 27605504

[23] Shamsi S, Suthar J. A revised method of examining fish for infection with zoonotic nematode larvae. Int J Food Microbiol. 2016;227: 13–16. 10.1016/j.ijfoodmicro.2016.03.023 27043384

[24] Winberg S, Lepage O. Elevation of brain 5-HT activity, POMC expression, and plasma cortisol in socially subordinate rainbow trout. Am J Physiol Regul Integr Comp Physiol. 1998;274(3): R645–R654. 10.1152/ajpregu.1998.274.3.R645 9530229

[25] Øverli Ø, Harris CA, Winberg S. Short-term effects of fights for social dominance and the establishment of dominant-subordinate relationships on brain monoamines and cortisol in rainbow trout. Brain Behav Evol. 1999;54(5): 263–275. 10.1159/000006627 10640786

[26] Hannes RP, Franck D, Liemann F. Effects of rank‐order fights on whole‐body and blood concentrations of androgens and corticosteroids in the male swordtail (*Xiphophorus helleri*). Z Tierpsychol. 1984;65(1):53–65.

[27] Enkel T, Gholizadeh D, und Halbach OVB, Sanchis-Segura C, Hurlemann R, Spanagel R et al Ambiguous-cue interpretation is biased under stress-and depression-like states in rats. Neuropsychopharmacol. 2010;35(4): 1008. 10.1038/npp.2009.204 PMC3055368 20043002

[28] Papciak J, Popik P, Fuchs E, Rygula R. Chronic psychosocial stress makes rats more ‘pessimistic’ in the ambiguous-cue interpretation paradigm. Behav Brain Res. 2013;256: 305–310. 10.1016/j.bbr.2013.08.036 23993861

[29] Iyasere OS, Beard AP, Guy JH, Bateson M. Elevated levels of the stress hormone, corticosterone, cause ‘pessimistic’ judgment bias in broiler chickens. Sci Rep. 2017;7(1): 6860 10.1038/s41598-017-07040-y 28761063 PMC5537245

[30] Sneddon LU. Evolution of nociception and pain: evidence from fish models. Philos T Roy Soc B. 2019;374(1785):20190290. 10.1098/rstb.2019.0290 PMC6790376 31544617

[31] Ingram BA, Gavine FM, Lawson P. Fish health management guidelines for farmed Murray cod Fisheries Victoria Research Report Series No. 32. 2019. ISBN1741466784.

[32] Domenici P, Lefrançois C, Shingles A. Hypoxia and the antipredator behaviours of fishes. Philos T Roy Soc B. 2007;362: 2105–2121. 10.1098/rstb.2007.2103 PMC2442856 17472921

